# Epsilon-Aminocaproic Acid in Liver Transplantation: A Three-Year, Retrospective Review

**DOI:** 10.4172/2155-6148.1000328

**Published:** 2013-06-27

**Authors:** Joseph R Whiteley, William R Hand, Harrison L Plunkett, Jason M Taylor, W David Stoll, Bethany J Wolf

**Affiliations:** Medical University of South Carolina, Charleston, USA

## Introduction

Improvements in surgical technique and transfusion strategies have decreased blood loss associated with liver transplantation in recent years [1]. However, blood loss and transfusion requirements are still an important risk factor for mortality during liver transplantation [2–4].

The antifibrinolytic drug epsilon-aminocaproic acid (EACA) was first used in liver transplantation in 1966 [5]. Inhibition of fibrinolysis during liver transplantation is a sensible transfusion strategy for several reasons. End stage liver disease is characterized by decreased clotting factors, resulting in a state of chronic fibrinolysis. Orthotopic liver transplantation causes tissue trauma and release of tissue plaminogen activator (t-PA). Tissue plasminogen activator is responsible for the conversion of plasminogen to plasmin, the main proteolytic enzyme of the fibrinolytic system [6]. In addition to the tissue trauma of the dissection phase, the anhepatic phase of surgery results in complete absence of clotting factors; which potentiates coagulopathy and contributes to blood loss. Following reperfusion of the graft, it is also common to see an acceleration of fibrinolysis [7].

Episilon-aminocaproic acid (EACA) is a synthetic lysine analog that competitively inhibits the conversion of plasminogen to plasmin. EACA also directly inhibits the proteolytic activity of plasmin. EACA’s antifibrinolytic properties are due to the prevention of plasmin formation, which is integral to fibrin lysis [8].

Despite widespread EACA use in liver transplantation to reduce fibrinolysis, few studies have demonstrated its efficacy. Prior studies have been limited by small sample size. Two retrospective studies, with fewer than twenty patients receiving EACA, showed no benefit [6]. There is one prospective, randomized-controlled study that compared EACA and tranexamic acid (TXA) to placebo [9]. In 124 patients, this study found a statistically significant reduction in transfusion requirements for patients receiving TXA only [9].

At our institution, EACA is used rather than TXA for antifibrinolytic therapy during orthotopic liver transplantation. One reason is cost, as TXA is approximately 20 times more expensive than EACA. Also the longstanding empiric use of EACA in liver transplantation is well established. Several studies examining aprotonin and TXA in liver transplantation used EACA for fibrinolysis rescue [10,11].

The goal of our study was to conduct the largest retrospective review of EACA use and transfusion requirements in orthotopic liver transplantation.

## Methods

We performed a retrospective review of all orthotopic liver transplants performed at the Medical University of South Carolina for the past three years. Of 238 liver transplants, 17 pediatric liver transplants (age < 18) were excluded, resulting in 221 cases for analysis. Our electronic anesthesia medical record system (PICIS, Wakefield, MA) and operative dictation reports were queried for the following data points: EACA usage (bolus, infusion, or bolus+infusion), red blood cell (RBC) transfusion, fresh frozen plasma (FFP) transfusion, Factor 7 usage, combined liver and kidney transplant, retransplants, pre-fibrinogen levels, post-fibrinogen levels, change in fibrinogen levels, MELD score, thrombus requiring reoperation, initial INR, and mortality (intraoperative and 30 day).

The primary aim was to determine whether EACA affected transfusion requirements. Secondary outcomes were effect of EACA use on fibrinogen level, operative time, and hepatic artery or portal vein thrombus formation. We examined the association between EACA and RBC and FFP transfusion volume; factor 7 administrations, presence of thrombosis, mortality, change in fibrinogen levels, and MELD score. Chi-square tests and Fisher’s exact test were used for comparing categorical variables. Student’s t-test or Mann-Whitney U test was used to examine associations between EACA usage and all continuous outcomes. All analyses were conducted in SAS v. 9.3 (SAS Institute, Cary, NC).

## Results

The data included 215 unique subjects and 221 procedures. The mean age of study participants was 55.3 years. A majority of the participants was male (63.3%). Eighty percent of study participants received EACA; 8.47% received EACA as a bolus, 23.7% received EACA as an infusion, and 67.8% received both a bolus and an infusion of EACA. Characteristics for the study population are shown in (Table 1).

There was not a significant difference in age or gender for subjects that received versus did not receive EACA. There also was not a significant difference between those that did and did not receive EACA in procedure duration, post-treatment fibrinogen levels, the proportion that underwent combined liver and kidney transplant, that received factor 7 or cell saver, that were re-transplants, or the proportion that died within 30 days of the procedure.

There were significant differences between those that received EACA and those that did not in regards to initial fibrinogen levels, change in fibrinogen levels, initial Tbili levels, initial INR levels, MELD score, and the proportion that received RBCs, FFP, and platelets. Subjects receiving EACA had significantly longer procedure duration relative to subjects that did not receive EACA (p=0.006). Subjects that received EACA had lower initial fibrinogen levels and a smaller change in fibrinogen levels (p<0.001 and p=0.003 respectively). Subjects that received EACA had higher Tbili, INR, and MELD score (p<0.001, p=0.003, and p<0.001 respectively). A significantly greater proportion of subjects that received EACA also received RBCs, FFP, and platelets (p=0.005, p<0.001, and p<0.001 respectively). Subjects that received EACA also had a smaller proportion of thrombus requiring reoperation, however the difference did not reach significance (p=0.070). Odds of receiving RBCs for subjects given EACA were 3.1 times the odds for subjects not receiving EACA (95% CI 1.38-7.04). Odds of receiving FFP for subjects given EACA were 8.1 times the odds for subjects not receiving EACA (95% CI 3.58-18.2). Odds of receiving platelets for subjects given EACA were 6.6 times the odds for subjects not receiving EACA (95% CI 2.89-14.1). The odds of a thrombus among subjects that received EACA were 0.38 times the odds of thrombus among subjects that did not receive EACA (95% CI 0.14-1.04).

### Transfusion requirements in non-EACA versus EACA groups

There were 44 subjects in the study that did not receive EACA and 177 subjects that did receive EACA. Of the 44 subjects that did not receive EACA, 33 subjects received RBCs (although one of these subjects received cell saver only and not packed RBCs). 159 of the 177 subjects that received EACA also received RBCs. 26 of the 44 subjects that did not receive EACA received FFP and 163 of the 177 subjects that received EACA also received FFP.

We compared RBC and FFP usage between the EACA and non-EACA groups using the Mann Whitney U test. Among all 221 subjects there was a significant difference in the amount of RBCs used for the EACA vs. no EACA group, with the EACA group receiving significantly more RBCs than the group that did not get EACA (p<0.001). The difference in amount of RBCs received was also significantly greater in the EACA group if we consider only those subjects that received RBCs (p<0.001). Across all patients, there was also a significant difference in the amount of FFP given between the EACA vs. non-EACA group, with the EACA group getting significantly more FFP (p<0.001). Again, this difference remained significant if we consider only the subgroup of patients that received FFP (p<0.001). The mean (+SE) and median for RBCs and FFP are reported in (Table 2).

### Comparing EACA usage outcomes controlling for MELD score

The MELD score was originally designed to gauge mortality while awaiting transplant. As such, it is a marker for disease severity. Given that patients with higher MELD scores are more likely to have worse prognosis and outcomes, we wanted to compare blood product usage between subjects that did and did not receive EACA while controlling for MELD score. Outcomes of interest included use of RBCs, FFP, and platelets, whether or not subjects had thrombus requiring reoperation, and mortality. We also compared the amount of RBCs and FFP received among patients that got these blood products.

Use of RBCs was significantly associated with MELD score, such that a 1 unit increase in MELD score was associated with a 9% increase in the odds of a patient receiving RBCs controlling for EACA usage (OR=1.09, 95% CI=1.03-1.16, p=0.004). However, EACA usage was not associated with receiving RBCs after controlling for MELD score (p=0.094). Subjects that received EACA had 7 times the odds of receiving FFP relative to subjects that did not receive EACA controlling for MELD score (OR=7.1, 95% CI=3.1-16.7, p<0.001). There was not a significant association between MELD score and whether or not a patient received FFP after controlling for EACA usage (p=0.357). Subjects that received EACA also had significantly greater odds of receiving platelets compared to those who did not receive EACA controlling for MELD score (OR=5.3, 95% CI=2.4-12.5, p<0.001). There was also a significant association between MELD score and whether or not a patient received platelets after controlling for EACA usage (p=0.006). There was not a significant association between receiving EACA and whether or not a subject had a clot after controlling for MELD score (p=0.117). There was a significant association between amount of RBCs and EACA usage after controlling for MELD score; with subjects that received EACA also receiving significantly more RBCs (p=0.003). Similarly, there was a significant association between amount of FFP received and EACA usage after controlling for MELD score; with subjects on EACA receiving significantly more FFP (p=0.006).

### Subgroup analysis of subjects with MELD ≤15 and subjects with MELD >15

#### MELD <15:

There were 91 subjects with a MELD score ≤ 15. The mean age of these participants was 55.4 years of age. A majority of the participants were male (65.9%). Seventy percent of study participants with MELD score ≤15 received EACA and of that 70%; 6.25% received EACA as a bolus, 28.1% received EACA as an infusion, and 65.6% received both a bolus and an infusion of EACA. Characteristics for subjects with MELD ≤ 15 are shown in (Table 3).

Among subjects with MELD score ≤ 15 there was not a significant difference in age or gender for subjects that received versus did not receive EACA. There also was not a significant difference between those that received versus did not receive EACA: in procedure duration, pre or post-treatment fibrinogen levels, change in fibrinogen levels, initial INR levels, initial creatinine levels, the proportion that underwent dual liver and kidney transplant, the proportion that died within 30 days of the procedure; those that received factor 7, cell saver, RBCs, had thrombus requiring reoperation, or those that were retransplants. There were significant differences between those that received versus did not received EACA: in amount of packed red cells transfused, MELD score, the proportion that received FFP, and the proportion that received platelets. Subjects that received EACA had higher MELD scores (p<0.001). Subjects receiving EACA also received a significantly greater volume of packed red cells (p=0.016). A significantly greater proportion of subjects that received EACA also received FFP and platelets (p<0.001 and p=0.006 respectively).

#### MELD >15:

There were 130 subjects with a MELD score >15. The mean age of these participants was 55.2 years of age. A majority of the participants were male (60.8%). Eighty-seven percent of study participants with MELD score >15 received EACA; and of that 87%, 9.73% received EACA as a bolus, 21.2% received EACA as an infusion, and 69.0% received both a bolus and an infusion of EACA. Characteristics for subjects with MELD >15 are shown in (Table 4).

Among subjects with MELD score >15 there was not a significant difference in age or gender for subjects that received versus did not receive EACA. There also was not a significant difference between those that did and did not receive EACA in procedure duration, post-treatment fibrinogen levels, initial INR levels, initial creatinine levels, the proportion that underwent dual liver and kidney transplant, the proportion that died within 30 days of procedure; that received factor 7, cell saver, RBCs, had thrombus requiring reoperation, or that were retransplants. There were significant differences between those that received EACA and those that did not in initial fibrinogen levels, change in fibrinogen, amount of packed red cells received, proportion that received FFPs, and the proportion that received platelets. Subjects that received EACA had lower initial fibrinogen levels and a smaller change in fibrinogen level pre versus post procedure (p=0.020 and 0.008 respectively). Subjects receiving EACA also tended to receive a larger volume of packed red cells compared to subjects that did not receive EACA (p<0.001). A significantly greater proportion of subjects that received EACA also received FFP and platelets (p=0.010 and p<0.001 respectively).

## Discussion

Our retrospective review of 221 adult orthotopic liver transplants did not show a reduction in transfusion requirements for patients receiving EACA. In our data set, the EACA group received significantly more RBCs, FFP, and platelets. An attempt was made to limit selection bias by performing a subset analysis of only recipients with MELD ≤ 15, the cut-off for non-exception transplantation. Despite this effort, our results show that the healthier patients with a MELD ≤ 15 in the EACA group still received twice the RBCs compared to those in the non-treatment group. These results are likely due to analysis bias, as the granularity of our data did not allow the authors to differentiate if EACA therapy was initiated after significant bleeding and resulting transfusion had already occurred. This potential bias notwithstanding, our results suggest an association between EACA administration and greater total amount and frequency of RBC, FFP, and platelet transfusion.

While transfusion requirements were greater in patients receiving EACA, it is important that the decrease in fibrinogen levels were significantly attenuated in the EACA group. Antifibrinolytic therapy has been previously shown to reduce fibrinolysis in liver transplantation [11]. Dalmau et al. showed decreased transfusion requirements in patients who received TXA and EACA, as well as an attenuated decline in fibrinogen levels [9]. The fact our EACA group’s fibrinogen level did not decrease as much as the non-EACA group, despite higher transfusion requirements; suggests EACA is effective in preventing fibrinolysis. There are no other studies that have examined fibrinogen levels following EACA use in liver transplantation. There is literature supporting decreased perioperative transfusion requirements in patients receiving EACA for spine surgery with an associated increase in fibrinogen level [12].

Thrombosis due to antifibrinolytic therapy has long been a concern in liver transplantation [13]. There have been a least 30 reported cases of thromboembolic events during liver transplantation. The majority of patients did receive antifibrinolytic therapy; however causality cannot be drawn from these case reports [14]. The relatively low incidence of thromboembolic events in liver transplantation makes it a challenge to power a prospective study [15]. While the retrospective design of our study has many limitations, it is noteworthy that incidence of thrombus was greater in the EACA group, approaching statistical significance (p<0.07).

## Conclusion

This is the largest retrospective study examining the effect of EACA use in orthotopic liver transplantation. Despite the limitations of retrospective reviews, our results call into question the utilization of EACA. Other than a modest improvement in fibrinogen levels, the use of EACA was associated with greater transfusion requirements. Given the likelihood of significant bleeding necessitating transfusion in orthotopic liver transplantation, further trials determining the optimal dose, timing, and choice of antifibrinolytics are needed. The lack of increased morbidity and mortality in our EACA group indicates empiric use does not likely increase risk to patients while we await prospective studies.

## Figures and Tables

**Table 1:** Characteristics of the study population.

Variable	All Subjects (N=221)		No EACA (N=44)		EACA (N=177)		p
	Mean ± SD	Median	Mean ± SD	Median	Mean ± SD	Median	
Age	55.3 ± 10.4	57.2	55.0 ± 11.9	58.6	55.4 ± 10.0	57.0	0.603
Duration (hours)	6.38 ± 1.80	6.06	5.81 ± 2.10	5.64	6.52 ± 1.69	6.17	0.006
Initial Fibrinogen (mg/dL)	225.4 ± 98.4	206	273.0 ± 102.8	263.5	213.5 ± 93.9	193	<0.001
Post Fibrinogen	188.4 ±52.0	180	200.5 ± 64.1	188	185.4 ± 48.3	178	0.148
Change in Fibrinogen	36.5 + 90.9	31.0	72.5 ±87.0	77.5	27.5 ± 89.8	19.0	0.003
Initial Creatinine	1.27 ± 1.20	1.00	1.27 ± 1.99	0.85	1.27 ± 0.91	1.00	0.054
Initial Tbili	6.23 ± 7.82	3.40	5.49 ± 8.94	1.8	6.41 ± 7.53	3.70	<0.001
Initial INR	1.72 ± 0.68	1.59	1.58 ± 0.56	1.45	1.75 ± 0.71	1.60	0.003
MELD score	17.5 ± 7.92	16.9	13.8 ± 8.63	12.1	18.4 ± 7.49	17.8	<0.001
Categorical Variables	n of N=221	% All Subjects	N of N=44	% No EACA	N of N=177	% EACA	p
Sex (% Male)	139	62.9%	29	65.9%	110	62.1%	0.729
RBC	190	86.0%	32	72.7%	158	89.3%	0.005
Cell Saver	17	7.69%	2	4.55%	15	8.48%	0.535
FFP	189	85.5%	18	59.1%	163	92.1%	<0.001
Platlets	119	53.9%	9	20.5%	110	62.2%	<0.001
Factor 7	18	8.14%	4	9.09%	14	7.91%	0.762
Liver & Kidney	19	8.60%	3	6.82%	16	9.04%	0.772
Retransplant	17	7.69%	3	6.82%	14	7.91%	1.000
Thrombus	19	8.60%	7	15.9%	12	6.78%	0.070
Mortality	6	2.71%	1	2.27%	5	2.73%	1.000

**Table 2:** Transfusion requirements in non-EACA versus EACA groups.

All Subjects							
Blood Product	No EACA			EACA			p
	N	Mean (SD)	Median	N	Mean (SD)	Median	
RBCs (ml)	44	899.5 (983.7)	600	177	2689.8 (2723.4)	1806	<0.001
FFP (ml)	44	872.2 (978.1)	600	177	2566.4 (2270.0)	1853	<0.001
Subjects that received blood product							
	N	Mean (SD)	Median	N	Mean (SD)	Median	p
RBCs (ml)	33	1236.9 (954.7)	1050	159	3044.6 (2716.4)	2100	<0.001
FFP (ml)	26	1399.8 (901.1)	1208	163	2786.9 (2231.6)	2100	<0.001

**Table 3:** Characteristics of the participants with MELD <15 by procedure.

Variable	No EACA (N=27)		EACA (N=64)		P
	Mean ± SD	Median	Mean ± SD	Median	
Age	57.5 ± 8.88	59.1	54.5 ± 9.79	56.1	0.172
Duration (hours)	5.79 ± 1.72	5.62	6.17 ± 1.40	5.93	0.278
Initial Fib	272.9 ± 79.7	276.0	246.2 ± 106.8	220.0	0.229
Post Fib	204.7 ± 68.5	194.0	183.0 ± 54.3	173.0	0.113
Change in Fib	68.2 ± 60.3	77.0	61.3 ± 83.8	51.0	0.661
Initial Creatinine	0.80 ± 0.23	0.80	0.80 ± 0.25	0.75	0.925
Initial Tbili	1.57 ± 1.09	1.40	3.59 ± 4.73	2.55	<0.001
Initial INR	1.37 ± 0.20	1.37	1.45 ± 0.22	1.45	0.085
RBCs	931.0 ± 1176	600.0	1871 ± 2262	1200	0.016
MELD score	8.29 ± 4.16	8.29	11.4 ± 3.38	12.68	<0.001
Categorical Variables	n of N=27	% No EACA	n of N=64	% EACA	P
Sex (% Male)	18	66.7%	42	65.6%	0.924
RBCs	18	66.7%	53	82.8%	0.089
Cell Saver	2	7.41%	7	10.9%	0.720
FFP	14	51.9%	57	89.1%	<0.001
Platlets	5	18.5%	32	50.0%	0.006
Factor 7	0	0.00%	2	3.13%	1.000
Liver & Kidney	1	3.70%	1	1.56%	0.508
Retransplant	2	7.41%	3	4.69%	0.631
Thrombus	5	18.5%	6	9.38%	0.292
Mortality	0	0.00%	2	3.13%	1.000

**Table 4:** Characteristics of the participants with MELD >15 by procedure.

Variable	No EACA (N=17)		EACA (N=13)		P
	Mean ± SD	Median	Mean ± SD	Median	
Age	51.0 ± 15.0	57.3	55.9 ± 10.2	57.3	0.207
Duration (hours)	5.83 ± 2.67	5.83	6.71 ± 1.81	6.33	0.041
Initial Fib	273.3 ± 134.3	239.0	195.0 ± 84.1	172.0	0.024
Post Fib	193.9 ± 57.7	175.0	186.7 ± 44.7	180.0	0.835
Change in Fib	79.4 ± 119.7	80.0	8.46 ± 87.76	1.00	0.008
Initial Creatinine	2.02 ± 3.09	1.30	1.54 ± 1.03	1.20	0.855
Initial Tbili	11.73 ± 12.07	6.60	8.02 ± 8.33	5.40	0.487
Initial INR	1.92 ± 0.77	1.72	1.92 ± 0.82	1.76	0.617
RBCs	896.1 ± 592.8	1050	3224 ± 2864	2400	<0.001
MELD score	22.60 ± 6.20	20.29	22.35 ± 6.18	20.8	0.893
Categorical Variables	n of N=17	% No EACA	n of N=113	% EACA	p
Sex (% Male)	11	64.7%	68	60.2%	0.721
RBCs	14	82.4%	105	92.9%	0.157
Cell Saver	0	0.00%	8	7.08%	0.596
FFP	12	70.6%	106	93.8%	0.010
Platlets	4	23.5%	78	69.0%	<0.001
Factor 7	4	23.5%	12	10.6%	0.225
Liver & Kidney	2	11.8%	15	13.3%	1.000
Retransplant	1	5.88%	11	9.73%	1.000
Thrombus	2	11.8%	6	5.31 %	0.281
Mortality	1	5.88%	3	2.65%	0.433
